# Measles Vaccine Virus RNA in Children More Than 100 Days after Vaccination

**DOI:** 10.3390/v11070636

**Published:** 2019-07-10

**Authors:** Jamie McMahon, Ian M Mackay, Stephen B Lambert

**Affiliations:** 1 Public Health Virology Laboratory, Forensic and Scientific Services, 39 Kessels Road, Coopers Plains QLD 4108, Australia; 2 Child Health Research Centre, The University of Queensland, 62 Graham Street, South Brisbane QLD 4101, Australia

**Keywords:** measles vaccine, measles virus, vaccines, measles, RT-PCR, vaccine safety, measles-mumps-rubella, RNA, persistence

## Abstract

Measles vaccines have been in use since the 1960s with excellent safety and effectiveness profiles. Limited data are available on detection of measles vaccine virus (MeVV) RNA in human subjects following vaccination. Available evidence suggests MeVV RNA can be identified up to 14 days after vaccination, with detection beyond this rare. In routine diagnostic testing, we used two real-time reverse transcription-polymerase chain reaction (RT-rPCR) assays targeting M and F genes to identify measles virus (MeV) and MeVV RNA. Confirmatory testing was performed with an N gene RT-rPCR, followed by sequence confirmation of RT-rPCR positives by semi-nested conventional RT-PCR assays targeting portions of the N, H, and L genes. We report detection and confirmation of MeVV RNA from the respiratory tract of 11 children between 100 and 800 days after most recent receipt of measles-containing vaccine. These novel findings emphasize the importance of genotyping all MeV detections and highlight the need for further work to assess whether persistent MeVV RNA represents viable virus and if transmission to close contacts can occur.

## 1. Introduction

Measles virus (MeV) is one of the most infectious pathogens of humans. It is highly transmissible with 90% of non-immune contacts exposed becoming infected; a high level of population immunity is required to interrupt transmission [1,2]. MeV typically causes a self-limited febrile illness, however, complications, often due to MeV induced immunosuppression, occur in up to 30% of cases, and can result in pneumonia, encephalitis, and, in rare cases, death [3,4].

Developed in the 1960s from clade A wildtype viruses, live attenuated measles vaccines have excellent safety and effectiveness profiles [2]. Use of the measles vaccine has contributed to substantial declines in child mortality and morbidity. Its use between 2000–2016 prevented an estimated 20.4 million deaths globally, a reduction of 84% in measles-associated deaths worldwide over the period [5].

Measles virus epidemiology is monitored using molecular typing, with eight clades designated A–H containing 24 recognised genotypes [6]. Following global vaccine use, wild genotype A is now considered inactive with genotype A detections exclusively vaccine-associated [6].

Measles has been well controlled in Australia for more than a decade, with elimination of endemic measles transmission likely to have occurred as early as the turn of the century [7]. In March 2014, Australia was certified by the World Health Organization (WHO) as having eliminated measles [8]. In an elimination setting it is important that effective surveillance for MeV is maintained [9]. Laboratory confirmation of all suspected cases presenting with a measles compatible clinical illness is critical to ensure a rapid public health response [10].

Information about the detection of measles vaccine virus (MeVV) and MeVV RNA in human clinical samples is limited. Here we report a series of extremely delayed MeVV RNA detections, identified by testing specimens submitted for diagnostic measles PCR, more than 100 days following vaccination in Queensland, Australia.

## 2. Materials and Methods 

Our nationally accredited, publicly-funded specialist public health virology laboratory (Forensic and Scientific Services) receives requests for routine diagnostic measles PCR directly from primary care physicians and hospitals, or via private pathology providers, in Queensland and northern New South Wales, Australia. Between 2013 and 2017, our laboratory was the only laboratory in Queensland routinely offering measles PCR testing. Detailed illness and medical history from individuals who had specimens submitted for testing were not routinely available.

The work reported in this paper was deemed to be a quality assurance activity as described in the National Statement on Ethical Conduct in Human Research, and was granted a waiver of ethics review by the Children’s Health Queensland Human Research Ethics Committee (HREC) [11]. The Forensic and Scientific Services Human Ethics Committee assessed this project as not requiring full HREC review, as it was not recognized as research, and is an audit of practice in accordance with the definition of research in the National Statement on Ethical Conduct in Human Research [11]. 

**Figure 1:**
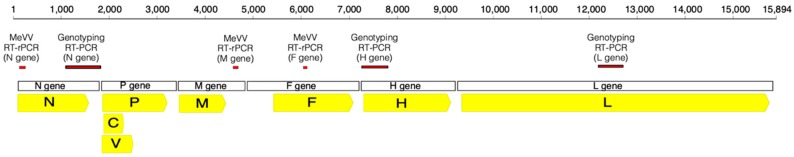
Schematic representation of the measles virus (MeV) genome highlighting the genes (open boxes), proteins produced (yellow arrows) and the diagnostic sequencing PCR assay targets (red boxes). The assays indicated are described in Table S1. The genome is drawn to scale and is based on MeV Edmonston strain (GenBank accession: AF266288). Higher resolution version is located at https://doi.org/10.6084/m9.figshare.8248082.

Upon receipt of specimens with a request for MeV PCR, total RNA was extracted using the Qiagen EZ1 Mini kit v 2.0 (Qiagen, Hilden, Germany). Five of the 90 µL elution was added to a measles virus (MeV) real-time reverse transcription-polymerase chain reaction (RT-rPCR) targeting the fusion (F) gene (Figure 1), designed to detect all MeV genotypes [12]. Nucleic acids were re-extracted from all MeV positive samples and testing was repeated using the MeV RT-rPCR. Additionally, the extracts from positive samples of patients with no known measles contact history, recent vaccination history, or at the request of the local public health unit, were further tested with a measles vaccine virus (MeVV) RT-rPCR targeting the matrix protein (M) gene designed specifically to detect MeVV strain, genotype A.

The primer, probes, cycling conditions and validation of the MeV F gene and the MeVV RT-rPCR assays have been described previously [12]. The MeV F gene RT-rPCR was used with a final primer concentration of 0.3 μM and probe concentration of 0.15μM. Primers and probe were used at a final concentration of 0.3 μM in the MeVV RT-rPCR. 

Confirmatory screening of all positives was performed with an additional novel MeV RT-rPCR, targeting the nucleoprotein (N) gene (Figure 1). Probes and primers are described in Table S1, with final primer concentration of 0.5μM and probe concentration of 0.2 μM.

All RT-rPCRs, sample extracts were reverse transcribed for 5 min at 50 °C (SuperScript^®^ III Platinum^®^ One-Step qRT-PCR Kit, Invitrogen, Carlsbad, CA, USA), incubated at 95 °C for 2 min, and then amplified using 40 cycles of 95 °C for 3 s and 60 °C for 30 s using either the Rotor-Gene Q or Rotor-Gene 6000 platform (Qiagen, Hilden, Germany).

Non-viral synthetic probe and primer controls were used for RT-rPCR assays, alongside no template controls (NTC), and negative extraction controls. No MeV was used as a positive control. A threshold cycle (C_T_) value of ≤40 indicated detection of MeV RNA whereas C_T_ values >40 were used to define a negative result. Virus culture is not performed as part of the MeV diagnostic process in our laboratory.

To confirm RT-rPCR positive samples we applied a semi-nested conventional RT-PCR requiring the generation of a 450-nucleotide (nt) fragment encoding the C-terminus of the N gene [12,13]. First-round RT-PCR amplification was performed after adding 5 µL of RNA extract to SuperScript™ III One-Step RT-PCR System with Platinum™ Taq DNA Polymerase (Invitrogen, Carlsbad, CA) mixes. A 30 min reverse transcription incubation at 55 °C and a 2 min inactivation at 94 °C preceded 40 cycles of 95 °C for 15 s, 60 °C for 30 s, and 68 °C for 60 s. A second round of amplification was performed by transferring 5 µL of 1:100 diluted Round 1 product into 15 µl Bioline MyFi™ Mix (BIOLINE GmbH, Luckenwalde, Germany) reactions followed by a 1 min denaturation step at 95 °C and 40 cycles of 95 °C for 15 s, 58 °C for 15 s, and 72 °C for 15 s.

Further confirmation of MeV positive samples was performed using two additional in-house RT-PCRs targeting the hemagglutinin (H) gene and the large protein (L) gene (Table S1, Figure 1) and followed the same method generating a 515-nt and 494-nt fragment, respectively. Amplification for round 1 for both H and L RT-PCRs was performed under the same conditions. A 30 min reverse transcription incubation at 55 °C and a 2 min inactivation at 94 °C followed by 40 cycles of 94 °C for 15 s, 50 °C for 30 s, and 68 °C for 30 s with a final incubation step of 68 °C for 5 min. Round 2 amplification for both H and L RT-PCRs consisted of a 1 min denaturation step at 95 °C preceding 40 cycles of 95 °C for 15 s, 52 °C for 15 s, and 72 °C for 15 s.

Amplicon analysis was performed with 0.2 µL Round 2 product using the QIAxcel (Qiagen, Hilden, Germany). For positive reactions, gel electrophoresis of the remaining amplicon was followed by purification of the excised band using QIAquick Gel Extraction Kit (Qiagen, Hilden, Germany). Dye terminator sequencing was performed on the CEQ8000 Genetic Analysis System (SCIEX, Framingham, MA, USA) using the GenomeLab DTCS Quick Start Kit (SCIEX, Framingham, MA).

Raw sequence data were cleaned, forward and reverse strands were aligned, and primer sequences removed. Sequences were aligned with the WHO designated MeV N and H reference strains and representative sequences available on GenBank for the L region. Phylogenetic analysis was conducted using Geneious Prime^®^ 2019.1.3 and phylogenetic trees were constructed with MEGA7 using neighbour-joining method and maximum likelihood method using a bootstrap analysis of 1000 replicates. Molecular characterisation of the samples was confirmed using the WHO Measles Nucleotide Surveillance (MeaNS) database genotyping tool for the N gene sequences [6].

Information on testing for other viruses by PCR performed by us or other diagnostic laboratories who initially received the specimens (either the same specimen or specimens collected around the time of the positive MeVV specimen) was collated. No sera were available for any of the cases to perform plaque reduction neutralization tests.

## 3. Results

Between 2013–2017, 9940 samples were received for routine diagnostic MeV PCR from Queensland and northern New South Wales. During this period, MeVV RNA was detected in 141 samples. 

In 2014, a local public health unit initiated a follow-up process for a patient who was MeV RT-rPCR positive and had no risk factors for measles infection, including no recent travel or contact history. The patient had presented with a mild clinical illness that, on follow-up, was felt to be not typical of MeV infection. Further investigation using a semi-nested conventional RT-PCR and sequencing confirmed MeVV RNA was present in this sample, collected 548 days after most recent measles vaccination (Table 1, case 4).

Having identified this unusual positive case, we sought out further MeVV positives in submitted specimens outside the previously documented timeframe of MeVV detection after vaccination. Between 2013–2017, MeVV strain RNA was detected in specimens from 11 children aged 18 to 45 months, more than 100 days (range: 101–784 days) following receipt of the most recent measles-containing vaccine (Table 1, Table 2). All 11 cases were followed up by local public health units for confirmation of vaccination dates recorded in the government immunisation register. Vaccines received included M-M-R II (Merck Sharp & Dohme Corp, West Point, Pennsylvania, US) (4 children), Priorix (GSK Biologicals, Rixensart, Belgium) (5 children), and Priorix-tetra (GSK Biologicals, Rixensart, Belgium) (2 children). Other MeVV detections during this period included 106 samples detected within 20 days after administration of a measles-containing vaccine. A further 10 samples were detected between 21–40 days, and the remaining 12 cases were detected between 41–100 days after vaccination (Table 3).

**Figure 2:**
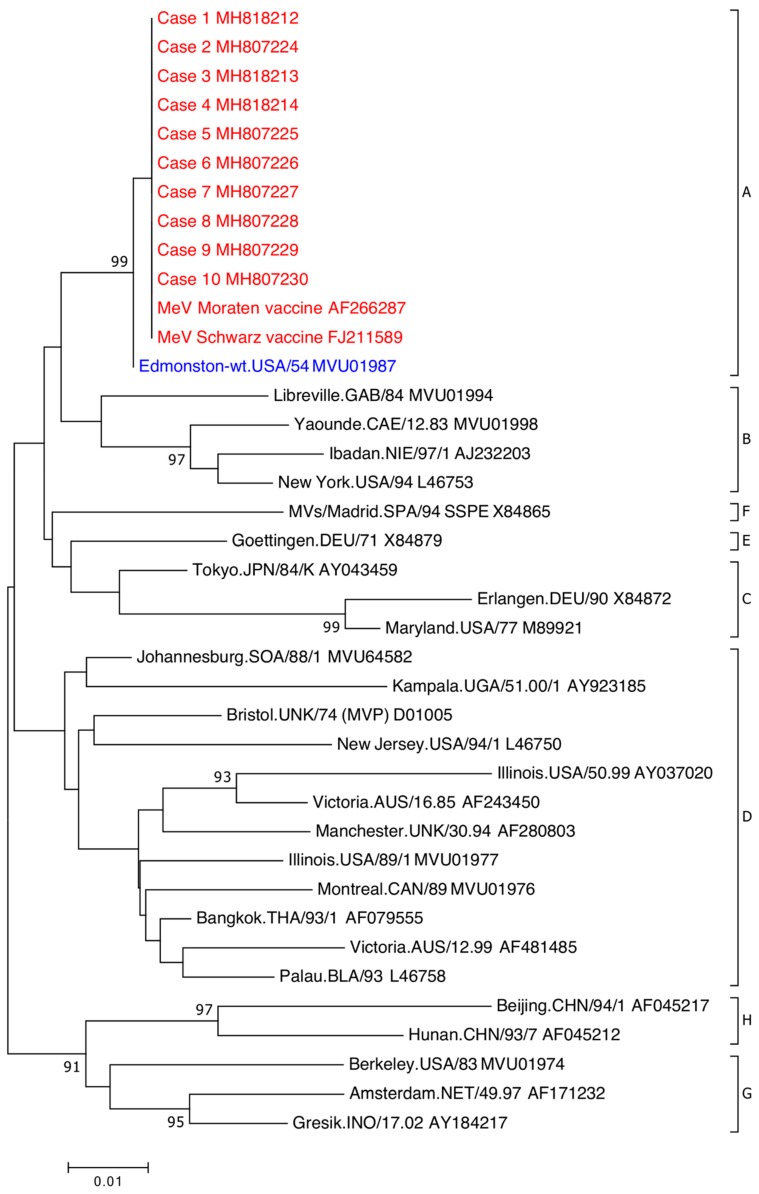
Phylogenetic analysis of MeV partial nucleoprotein (N)-gene sequences. The sequenced region is the World Health Organization (WHO)-recommended 450-nt nucleoprotein (N) gene fragment encoding the carboxyl-terminal of the gene. WHO MeV reference strain sequences include GenBank accession numbers and by eight clades designated A–H. Clade A MeVV sequences associated with patients (red) are indicated by case number and associated GenBank accession number. Clade A wildtype MeV (blue) indicated by WHO reference strain and GenBank accession number. Phylogenetic analysis was used the neighbour-joining method. Maximum composite likelihood methods was performed in MEGA7 with a bootstrap analysis of 1000 replicates. The percentage of replicate trees in the bootstrap test (1000 replicates) greater than 85 are shown next to the branches. A higher resolution version is located at https://doi.org/10.6084/m9.figshare.7649063.

All 11 cases were detected using the MeV F gene RT-rPCR and MeVV RT-rPCR (Table 2). Case 4 was not detected initially using the MeVV RT-rPCR, however upon repeat testing, it was detected (MeVV RT-rPCR C_T_ value: 38). Ten cases confirmed by sequence analysis of the WHO-recommended 450nt N gene fragment were determined to be genotype A on the MeaNS database (Figure 2). The remaining case, case 11, was initially not confirmed using the N gene RT-PCR. Upon repeat testing, a faint DNA fragment was present but could not be sequenced. The weak signal was likely due to the low level of RNA present in this extract (F gene RT-rPCR C_T_ value: 39). The presence of MeVV in case 11 was confirmed by sequencing the L gene RT-PCR (Figure S2).

Eight of nine cases tested were positive in the N gene RT-rPCR; cases 1 and 10 were not tested due to insufficient extract available. Eight cases were further confirmed by sequence on the H gene RT-PCR (Figure S1). Case 7 produced a faint DNA fragment which could not be sequenced. Sequencing of the L gene RT-PCR confirmed the presence of MeV in eight cases, case 7 and case 8 produced a faint DNA fragment but could not be sequenced (Figure S2). All cases sequenced on the N gene and the L gene showed 100% homology, however there was a single point mutation in case 4 at nucleotide position 404 of the H gene which generated a nonsynonymous change from glycine to aspartic acid (G404D; Figure S1).

MeVV RNA was detected from nasopharyngeal (7), nasal (2), and unspecified respiratory swab sites (2). Five children had concurrent non-MeV respiratory virus detections; respiratory syncytial virus (1), respiratory syncytial virus and human parainfluenza virus 3 (1), adenovirus (2), and human metapneumovirus (1) (Table 4).

Of the 11 patients, four had urine collected concurrently. MeV RNA could not be detected in any of these samples. Case 9, who had MeVV RNA detected 110 days post-vaccination had both a throat and a nasal swab collected. The nasal swab was positive, but the throat swab was not. 

From these 11 cases, extracts from six with the lowest C_T_ values and sufficient extract remaining were sent to the WHO Regional Measles Reference Laboratory at the Victorian Infectious Diseases Reference Laboratory (VIDRL), Melbourne, Australia, for external confirmatory testing. Cases 5, 6, 8, and 9 were all positive for MeV RNA on a previously described measles RT-rPCR assay and typed as genotype A (vaccine strain) by RT-rPCR [14]. Cases 2 and 3 were not confirmed by VIDRL.

## 4. Discussion 

In response to routine clinical requests for MeV PCR testing, we found MeVV RNA in submitted respiratory specimens between 100 and 800 days after most recent vaccination date from 11 children over a period of five years. We confirmed MeVV RNA detection using five additional unique targets including MeV RT-rPCR, as well as an MeVV-specific RT-rPCR assay. This was followed by use of three semi-nested conventional RT-PCR and nucleotide sequencing regions of the N, H, and L genes. Findings of this nature in any human clinical sample type, so long following documented vaccination, have not been described previously and require studies at other sites to confirm. 

Following natural respiratory tract infection, MeV spreads to the local lymphoid tissue via infected lymphocytes, macrophages, and dendritic cells [15]. Virus is exported systemically to a wide range of organs through infected cells of the myeloid lineage [15]. MeV is cleared from the site of replication by a functioning adaptive immune response [16]. However, MeV can persist in lymphoid tissue for six months after infection, where it may contribute to the maturation of the immune response to infection [16,17,18]. Failure of early innate immunity can lead to inhibition of the type I interferon response, empowering spread and delaying clearance [16]. Our findings in the respiratory tract together with this knowledge of persistence suggest that local lymphoid tissue may be an important target for future prospective study.

MeV RNA detection has been described several months after infection and from a range of tissues [19,20]. Persistent, often mutated, MeV infection causes rare, sometimes fatal, complications, including measles inclusion body encephalitis and subacute sclerosing panencephalitis, many years post-infection [21,22]. MeV RNA, typing not further described, was detected in ileal biopsies from two children at least six months following receipt of their most recent measles-containing vaccine, but no respiratory sites were tested [23]. Delayed MeV detection has been reported in non-respiratory tissues: One paper described the presence of MeV RNA in lymphocytes of healthy people who had a wildtype infection up to 30 years prior [24]. However, sequence analysis in this study suggested recent, asymptomatic infection of genotype D5, the circulating wild-type MeV strain at the time of the study, rather than MeV persistence. MeV RNA has been detected in bone marrow aspirates taken for the diagnosis of malignant bone diseases [25]. MeV RNA has been found in autopsy tissue from apparently healthy people using PCR with detections in the brain, lung, liver, kidney, and spleen (8–20% detection prevalence) [26]. Sequence analysis of the partial N, H, and L genes in our study demonstrated 100% homology of all cases to MeVV except for one case with a nonsynonymous substitution in the H gene (G404D), raising the possibility of further substitutions in regions not sequenced that may play a role in persistence.

MeV persistence following vaccination has been evident in immunosuppressed patients. Being a live virus vaccine, administration of the measles vaccine is contraindicated for patients with immunodeficiencies. Individuals with HIV are still recommended to receive a measles-containing-vaccine unless there is indication of immunosuppression at time of vaccination [27]. There is one report of an unknown immunodeficiency leading to measles inclusion body encephalitis following vaccination [28]. Human IFNAR2 deficiency has also been linked to a fatal encephalitis with persistent measles IgM following vaccination with a measles-containing-virus [29].

Data on MeVV detection in human clinical samples are limited: This may be because specific testing for MeVV is uncommon, detection of MeVV is uncommon, symptoms are not associated with presence of MeVV RNA, or a combination of these. Unlike wildtype MeV, MeVV sequences are not submitted routinely to the WHO Measles Nucleotide Surveillance database [6]. It is not unusual to find MeVV detection up to 14 days after vaccination [30,31,32]. There is one case report of detection from the nasopharynx five weeks post-vaccination [33]. Vaccine-associated genotype A has also been previously detected in urine 25 days following vaccination [34].

Our description of this small case series is likely made possible for several reasons: Enhanced surveillance involving increased requests for testing in an elimination setting; the application of increasingly sensitive PCR assays to many thousands of specimens; and the ability to rapidly detect and differentiate wildtype MeV from MeVV.

External evaluation of a portion of extracts at the Australian WHO Regional Measles Reference Laboratory confirmed four of our cases as MeVV positive; the two remaining cases were negative. It is worth noting initially that even for specimens that tested positive in both laboratories, C_T_ values were high, and it is likely that we are identifying RNA close to the limit of detection. In this setting, it is unsurprising there is inter-laboratory variability around some, but by no means a majority, of dual tested specimens. Whilst not likely to be a major issue, transit and subsequent laboratory processes at the receiving laboratory may lead to RNA loss. For the two samples with incongruent results, the findings were validated in our laboratory using a variety of approaches, including sequence data from three distinct genetic regions (N, H, and L genes) and real-time PCRs targeting the N, M, and F genes.

False positive results and laboratory contamination were considered and rejected as plausible explanations for our findings. Our nationally accredited public health virology laboratory undergoes regular audits and maintains molecular assays that undergo extensive validation. Assay performance is regularly reviewed to maintain accreditation for diagnostic testing, achieving nationally mandated standards of quality. This is to ensure we consistently produce reliable and meaningful data for our clinical clientele. Our laboratory uses a unidirectional workflow, within a PC3 laboratory for all PCR testing. The testing process is distributed across multiple rooms, with nucleic acid extraction and sample loading rooms under positive pressure to prevent contamination. Amplification and post-PCR analysis rooms are kept under negative pressure within the PC3 laboratory to contain amplicons. All work is performed using dedicated personal protective equipment, instruments, and reagents. Samples are handled in Class II biological safety cabinets and regular equipment maintenance and decontamination is undertaken. Sequencing is conducted in another physically separate and dedicated post-PCR area. Our laboratory processes undergo due diligence to ensure contamination does not occur, including the use of non-viral synthetic positive controls, no-template and negative controls, and the confirmation of any positives by re-extraction and testing. MeVV is not cultivated or used as a positive control in our laboratory and time between MeVV routine detections and our reported cases indicate no pattern of laboratory contamination. To further eliminate the chance of PCR-contamination, we used PCR assays located in five genetically distinct targets. False positive MeVV detections in which a non-circulating virus (Clade A) could be identified among these 11 cases identified over five years, with no common geographical region, was considered an extremely unlikely event.

We report these findings as an initial communication but recognise the limitations in our work. Firstly, we have reported data on only 11 episodes of MeVV RNA detection. These specific cases were subjected to further analysis due to the extreme period between detection and last recorded vaccination. From these 11 cases it is not possible to determine if MeVV RNA may be intermittently detectable in some or most vaccinated individuals, or whether we are reporting a truly rare event. Also, due to small case numbers, it is unclear if these detections are specifically associated with concurrent presence of other respiratory viruses or the cases could have unidentified immunodeficiencies causing persistence of MeVV. Finally, detection of MeVV RNA alone does not allow for any assessment of whether infectious virus is present.

We acknowledge that MeV vaccination still remains a safe and highly effective way of preventing the spread of MeV and our findings in no way contradict data on the effectiveness of the MeV vaccine. Importantly, our findings should not be used as a reason for leaving anyone unvaccinated against measles.

Further work is required to expand on our findings. This work includes assessing whether the MeVV RNA detected derives from viable virus or an RNA transcription process that produces non-infectious or no virus. If a live virus is produced it will be important to assess if transmission to close contacts occurs. To date, there is no documented evidence of human-to-human transmission of MeVV [35]. These further details are required to understand the significance of our findings, and their implications for immune boosting, disease control, and reaching and maintaining WHO measles elimination targets. Complete genome sequencing, requiring fresh clinical material, is also required to determine if there are further point mutations that may have developed that could lead to persistence. Our findings also highlight the importance of genotyping all MeV detections, not only to provide laboratory evidence of the absence of endemic MeV circulation, but to document the prevalence and detailed epidemiology of MeV clade A detection.

## Figures and Tables

**Table 1:** Details of cases with measles vaccine virus detection more than 100 days post-vaccination.

Case	Age, Sex	Days Post-Vaccination	Swab Site	Request Notes	Vaccine ^§^	Concurrent Detection/s ^ǁ^	Designated Genotype (N Gene RT-PCR ^‡^)
1	23mo, F	218	NP	Third day high fevers, rash appearing	MCV1 Priorix	HMPV	Genotype A
2	17mo, F	142	NP	Query measles	MCV1 M-M-R II	NT	Genotype A
3	25mo, M	345	Nasal	In Europe two weeks before illness	MCV1 Priorix	RSV	Genotype A
4	30mo, F	548	NP	Viral infection, lower lobe consolidation	MCV1 Priorix	AdV	Genotype A
5	16mo, F	125	Swab *	Rash, fever	MCV1 M-M-R II	RSV HPIV-3	Genotype A
6	16mo, M	147	NP	No notes	MCV1 M-M-R II	NT	Genotype A
7	33mo, M	471	NP	No notes	MCV2 Priorix-tetra	NT	Genotype A
8	15mo, M	101	NP	Non-itchy, red throat, whole-body rash, possible Koplik spots	MCV1 Priorix	ND	Genotype A
9	16mo, F	110	Nasal	Fever, rash. Rubella contact	MCV1 M-M-R II	NT	Genotype A
10	17mo, F	139	Swab *	Viral rash on face	MCV1 M-M-R II	AdV	Genotype A
11	45mo, M	784	NP	No notes	MCV2 Priorix-tetra	NT	Insufficient RNA

AdV = adenovirus; F = female; HMPV = human metapneumovirus; HPIV-3 = parainfluenza virus type 3; M = male; MCV1 = first dose of measles-containing-vaccine; MCV2 = second dose of measles-containing-vaccine; mo = months old; NP = nasopharyngeal swab; NS = no specimen collected; NT = not tested; RSV = respiratory syncytial virus. * swab site not recorded; † samples run in duplicate; ^§^ vaccine dose number and vaccine product received most recently prior to MeVV detection; ^ǁ^ routine respiratory virus PCR diagnostic testing performed in referring laboratories; ‡ genotype designated by World Health Organization (WHO) Measles Nucleotide Surveillance (MeaNS) database.

**Table 2:** Summary of MeV results.

Case	Days Post-Vaccination	MeV F Gene RT-rPCR (C_T_ Value) ^†^	MeVV RT-rPCR (C_T_ Value) ^†^	MeV N Gene RT-rPCR (C_T_ Value) ^†^	N Gene RT-PCR ^‡^	L Gene RT-PCR ^‡^	H Gene RT-PCR ^‡^	Designated Genotype (N Gene RT-PCR ^‡^^∂^)	Urine MeV F Gene RT-rPCR
1	218	33.21 32.86	34.12 33.29	NT	DET	DET	DET	Genotype A	ND
2	142	38.20 37.62	39.30 ND	39.14 38.10	DET	DET	ND	Genotype A	NS
3	345	37.11 36.87	38.11 ND	38.60 37.33	DET	DET^α^	DET	Genotype A	ND
4	548	36.40 37.24	ND ^*^ 38.93 *	38.93 ND	DET	DET	DET	Genotype A	NS
5	125	34.09 34.22	36.64 36.50	36.98 36.62	DET	DET	DET	Genotype A	NS
6	147	33.90 ND	35.22 ND	36.78 36.65	DET	DET	DET	Genotype A	ND
7	471	37.47 38.51	39.13 39.46	ND 39.21	DET	ND	DET^α^	Genotype A	ND
8	101	39.62 ND	38.82 ND	33.58 33.46	DET	DET ^α^	DET	Genotype A	NS
9	110	32.72 33.37	35.34 35.28	35.82 34.71	DET	DET	DET	Genotype A	NS
10	139	38.33 ND	39.30 ND	NT	DET	DET	DET	Genotype A	NS
11	784	39.62 ND	ND 38.82	39.41 39.88	DET^α^	DET	ND	Insufficient RNA	NS

C_T_ = threshold cycle; DET = detected; ND = not detected (C_T_ > 40); NP = nasopharyngeal swab; NS = no specimen collected; NT = not tested; RT-rPCR = real-time reverse transcription PCR; † samples run in duplicate; ‡ RT-PCR = conventional RT-PCR; ^α^ DNA fragment detected but not able to be sequenced; * Sample initially tested not detected, but detected upon repeat testing; ^∂^ genotype designated by WHO Measles Nucleotide Surveillance (MeaNS) database. MeV = measles virus; MeVV = measles vaccine virus.

**Table 3:** Count of measles vaccine virus detections and days from most recent measles-containing vaccine.

DAYS SINCE LAST MCV *	NUMBER OF MEVV CASES
0–19	106
20–39	10
40–59	5
60–79	4
80–100	3
>100	11
UNKNOWN	2

* MCV = measles-containing vaccine.

**Table 4:** Results from other PCR testing performed on respiratory specimens in referring laboratories using the same specimen or a specimen collected around the time of the positive measles specimen.

Case	PCR Testing Performed	Detections
1	RSV, IFAV, IFBV, HPIV-1, HPIV-2, HPIV-3, HMPV, RV, AdV	HMPV
2	Not tested for other viruses	NT
3	RSV, IFAV, IFBV, HPIV-1, HPIV-2, HPIV-3, HMPV, RV, AdV	RSV
4	RSV, IFAV, IFBV, HPIV-1, HPIV-2, HPIV-3, HMPV, RV, AdV	AdV
5	RSV, IFAV, IFBV, HPIV-1, HPIV-2, HPIV-3, HMPV, RV, AdV	RSV, HPIV-3
6	RSV, IFAV, IFBV, HPIV-1, HPIV-2, HPIV-3, HMPV, RV, AdV	ND
7	Not tested for other viruses	NT
8	RUBV *, RSV, IFAV, IFBV, HPIV-1, HPIV-2, HPIV-3, HMPV, RV, AdV	ND
9	RUBV *	ND
10	RSV, IFAV, IFBV, HPIV-1, HPIV-2, HPIV-3, HMPV, RV, AdV	AdV
11	Not tested for other viruses	NT

AdV = adenovirus; HPIV-1 = human parainfluenza virus type 1; HPIV-2 = human parainfluenza virus type 2; HPIV-3 = human parainfluenza virus type 3; HMPV = human metapneumovirus; IFAV = influenza A virus; IFBV = influenza B virus; ND = other viruses not detected; RSV = respiratory syncytial virus; RUBV = rubella virus; RV = rhinovirus; * rubella virus PCR testing performed in our laboratory; NT = not tested for other viruses.

## Supplementary Materials

The following are available online at https://www.mdpi.com/1999-4915/11/7/636/s1, Figure S1: Phylogenetic analysis of MeV partial hemagglutinin (H) gene sequences, Figure S2: Phylogenetic analysis of MeV partial large gene (L) gene sequences, Table S1: Primers and probes for MeV RT-rPCR and RT-PCR.
