# Supplementary material for: Measles Vaccine Virus RNA in Children More Than 100 Days after Vaccination
Source: Viruses. 2019 Jul 10;11(7):636. doi: 10.3390/v11070636 (PMC6669751; doi:10.3390/v11070636)
Supplement: Supplementary file 1 [file viruses-11-00636-s001.zip › supplmentary/Figure S1.pdf]

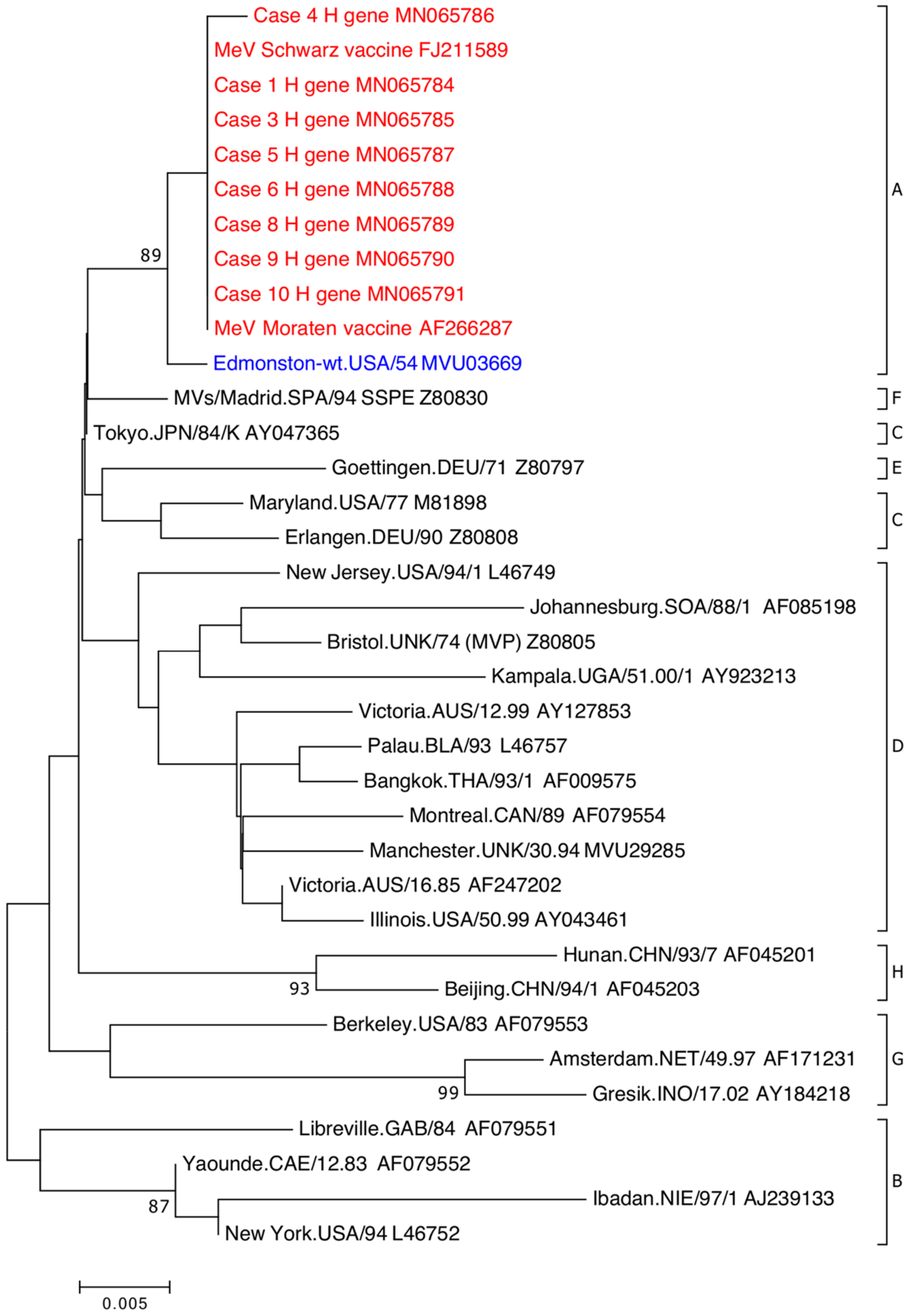

Figure S1: Phylogenetic analysis of MeV partial hemagglutinin (H) gene sequences.

Partial sequence generated using the H gene RT-PCR. WHO MeV reference strain sequences include GenBank accession numbers and the eight clades are designated A–H. MeVV sequences associated with patients and vaccine strain genotype A (red) viruses are indicated by case number and associated GenBank accession number. Wildtype genotype A reference strain is indicated in blue. Phylogenetic analysis used the Neighbor-Joining and Maximum Composite Likelihood methods and was performed in MEGA7 with a bootstrap analysis of 1000 replicates. The percentage of replicate trees in the bootstrap test (1000 replicates) greater than 85 are shown next to the branches. A higher resolution version is located at https://doi.org/10.6084/m9.figshare.8248139
