# Supplementary material for: Measles Vaccine Virus RNA in Children More Than 100 Days after Vaccination
Source: Viruses. 2019 Jul 10;11(7):636. doi: 10.3390/v11070636 (PMC6669751; doi:10.3390/v11070636)
Supplement: Supplementary file 1 [file viruses-11-00636-s001.zip › supplmentary/Table S1.docx]

Table S1: Primers and probes for MeV RT-rPCR and RT-PCR

| Assay | Primer or probe | 5’-3’ nucleotide sequence | Genomic positions* | Reference |
| --- | --- | --- | --- | --- |
| MeV F gene RT-rPCR | Measles MGB FP | GCTCAAATTGCTCAGATACTATACAGAAA | nt 6066–6094 | McMahon, et al. [12] |
|  | Measles MGB RP | GCAGATATGGGGTCCCGTAA | nt 6137–6118 |  |
|  | Measles MGB Probe | FAM-CCTGTCATTATTTGGCC-MGBNFQ | nt 6096–6112 |  |
| MeV N gene RT-rPCR | Measles N For | CAAGGACAAACCACCCAT | nt 149–166 | This study |
|  | Measles N Rev | TCCTCAATYACCACYCGATC | nt 253–234 |  |
|  | Measles N Probe | FAM-ACATCDGGATCCGGTGGRGC-BHQ1 | nt 168–187 |  |
| MeVV RT-rPCR | Measles F 4729 Vac | AAACCCCCAGCAATTGGAA | nt 4729–4747 | McMahon, et al. [12] |
|  | Measles R 4795 Vac | GGTCACCTCGGTCGCTTGT | nt 4813–4795 |  |
|  | Measles Probe 4757 | FAM-CCCTCTTCCTCAACACA-MGBNFQ | nt 4757–4773 |  |
| N gene RT-PCR | MVF1 | TACCCTCTGCTCTGGAGCTATGCC | nt 1092–1115 | Chibo, et al. [13] |
|  | MVB1 | AACAATGATGGAGGGTAGGCG | nt 1736–1716 |  |
|  | MVF2 | GATGGTAAGGAGGTCAGCTGG | nt 1208–1228 |  |
| H gene RT-PCR | MeV H For R1 | AGAYCATCCRCAATGTCACC | nt 7259–7278 | This study |
|  | MeV H Rev | CAGCWAGGAACTGRTTGGTT | nt 7817–7798 |  |
|  | MeV H For R2 | CCGRATAAATGCCTTYTACA | nt 7288–7307 |  |
| L gene RT-PCR | MeV L For | CCCTTACTCAGCWAATCTYGT | nt 12224–12244 |  |
|  | MeV L Rev R1 | GCAGATGACACATGTYTCAT | nt 12773–12754 | This study |
|  | MeV L Rev R2 | CTAGYACRTCAGGGACYTCA | Nt 12723–12704 |  |

FAM = 6-FAM (Fluorescein); MGBNFQ = minor groove binder, nonfluorescent quencher; BHQ1= Black Hole Quencher 1; RT-rPCR = real-time reverse transcription PCR; RT-PCR = conventional RT-PCR; nt = nucleotide

*nucleotide position based on Edmonston MeV strain, GenBank accession AF266288
